# Native Plant Responses and Elemental Accumulation in Mining and Metallurgical Mediterranean Ecosystems

**DOI:** 10.3390/plants14172646

**Published:** 2025-08-25

**Authors:** Eleni G. Papazoglou, Hamza Zine, Panayiotis Trigas, Małgorzata Wójcik, Jaco Vangronsveld

**Affiliations:** 1 Laboratory of Systematic Botany, Department of Crop Science, Agricultural University of Athens, 75 Iera Odos, 11855 Athens, Greece; trigas@aua.gr; 2 Geology and Sustainable Mining Institute (GSMI), Mohammed VI Polytechnic University, Ben Guerir 43150, Morocco; zine.hamza@um6p.ma; 3 Department of Plant Physiology and Biophysics, Institute of Biological Sciences, Maria Curie-Skłodowska University, 19 Akademicka, 20-033 Lublin, Poland; mwojcik@poczta.umcs.lublin.pl (M.W.); jaco.vangronsveld@uhasselt.be (J.V.); 4 Centre for Environmental Sciences, Hasselt University, Agoralaan Building D, 3590 Diepenbeek, Belgium

**Keywords:** phytoremediation, phytostabilization, native flora, bioaccumulation, heavy metals, polluted sites

## Abstract

Mining and metallurgical activities negatively impact ecosystems due to the release of potentially toxic elements (PTEs). This study assesses PTE pollution and accumulation in native plant species that have spontaneously colonized a historical mining site (Michaly, site A) and a nearby metallurgical smelter site (Varvara, site B) on the Lavreotiki Peninsula, Attika, Greece. Soils were analyzed for As, Cd, Co, Cr, Cu, Fe, Mn, Ni, Pb, Sb, and Zn. A total of 89 native plant taxa across 28 families were identified. The aerial parts from dominant species were analyzed for PTE concentrations, and bioconcentration factors (BCFs) were calculated. One-way ANOVA and principal component analysis (PCA) using R were used for statistical evaluation. Soils at both sites showed elevated As, Cd, Cr, Cu, Ni, Pb, Sb, and Zn; Mn was high only at site B, while Co and Fe remained at background levels. Several plant species, especially at Michaly, had elevated concentrations of As, Cd, Co, Cr, Fe, Pb, Sb, and Zn in their aerial parts. BCFs indicated general PTE exclusion from aerial parts, particularly at site B. Native vegetation on these contaminated sites shows resilience and PTE exclusion, highlighting their potential for phytoremediation, especially phytostabilization, and ecological restoration in similarly polluted Mediterranean environments.

## 1. Introduction

Since the industrial revolution, mining and metallurgical activities have become significant sources of anthropogenic soil pollution worldwide, particularly in regions with a long history of metal ore mining [1,2]. A key issue associated with mining operations is the mismanagement or excessive accumulation of large surface deposits of tailings containing high concentrations of potentially toxic elements (PTEs), both during and after mining activities, often without any form of remediation [3,4]. These elements include metals such as zinc (Zn), lead (Pb), cadmium (Cd), copper (Cu), uranium (U), as well as metalloids like germanium (Ge), antimony (Sb), arsenic (As), and bismuth (Bi). At elevated concentrations, these elements can disrupt physiological and biochemical processes in plants, animals, humans, and soil microorganisms [5]. According to Gul et al. [6], many PTEs are particularly mobile, potentially bioavailable, persistent, and pose a high risk of bioaccumulation in food chains.

Despite the high concentrations and toxicity of PTEs in polluted environments, some plant species have adapted to survive and even flourish in such areas [7,8]. These plants, known as metallophytes [9,10], have developed various mechanisms to either exclude PTEs or tolerate elevated levels within their tissues. Numerous studies have highlighted a range of morphological, anatomical, physiological, biochemical, and genetic strategies that plants employ to adapt to high concentrations of PTEs, often inducing enzymatic and non-enzymatic antioxidative mechanisms to mitigate the toxic effects [11,12,13,14,15]. These resilient plant species hold potential for phytoextraction, which aims to remove PTEs from polluted soils, thereby reducing soil toxicity and contributing to the restoration of ecosystem services [16,17]. Alternatively, they can also contribute to the stabilization of pollutants in the soil, preventing their spread to adjacent areas, surface water, and groundwater, thereby reducing risks to environmental and human health [18,19,20].

Lavreotiki, located southeast of Athens, has a long-standing history of intensive mining and metallurgical activities, primarily focused on extracting Pb and silver (Ag) from carbonate and sulphide ores [21,22,23]. These activities, which date back to ancient times and continued intermittently until the 1980s, have resulted in extensive waste stockpiles in the surrounding area and widespread pollution across the Lavreotiki Peninsula [24,25,26,27,28,29]. The mining sites and tailings from mineral processing in the area are rich in PTEs. Specifically, regions such as Thorikos, known for their historic Ag-Pb mines, contain acid-generating materials that serve as persistent sources of soil and water pollution [30,31]. Kontopoulos et al. [32] highlighted the geochemical and environmental characteristics of the waste generated by mining and metallurgical activities in this region. This situation underscores the need for targeted and sustainable remedial measures to protect the well-being of local populations and to prevent the spread of PTEs to adjacent natural ecosystems.

The main objective of this study was to assess the potential of native Mediterranean plant species, naturally colonizing soils historically impacted by mining and metallurgical activities, to serve as candidates for phytoremediation and ecological restoration. These regions, where spontaneous vegetation has adapted to extremely high levels of potentially toxic elements over decades, centuries, or even millennia, represent valuable natural “living laboratories” for identifying metal-tolerant species. Specifically, this study aimed to (i) evaluate the concentrations of PTEs (As, Cd, Co, Cr, Cu, Fe, Mg, Mn, Ni, Pb, Sb, and Zn) in soils and corresponding plant tissues, (ii) analyze interspecific differences in elemental accumulation patterns across sites, and (iii) identify native species with functional traits suitable for phytotechnologies under Mediterranean climatic conditions.

## 2. Results and Discussion

### 2.1. Characterization and PTE Concentration in Soil

Soil texture analysis classified both sites as sandy loam, with some variation in clay and silt content between the two locations (Table 1). Both soils exhibited slightly alkaline pH values and differed in organic matter content, which was notably higher at site B. These characteristics reflect moderate variability in soil properties between the mining (site A) and metallurgical (site B) areas.

The range of total concentrations of PTEs in the soils at sites A and B, as well as the diethylenetriaminepentaacetic acid (DTPA) extractable fractions are presented in Table 2. The total concentrations of Co and Fe in the soils at both study sites were low and remained within the natural background levels specified in Table 2. However, the total concentrations of As, Cd, Cr, Cu, Mn, Ni, Pb, Sb, and Zn in the soils were elevated at both sites compared to the threshold values set by the European Soil Directive (86/278/EEC) [33]. For elements not covered by the directive, the comparison was made against natural background levels reported by Kabata-Pendias [34].

Iron is a naturally abundant soil element, often reaching up to 100,000 mg kg^−1^ depending on geological conditions. Although present in high concentrations, Fe is generally non-toxic and is essential for plant metabolic functions. In this study, Fe concentrations were within normal background levels. As a mesonutrient, required in quantities between macronutrients and micronutrients, Fe was effectively regulated by the native species. The absence of deficiency or toxicity symptoms suggests that these plants maintain nutrient homeostasis under the given site conditions. According to Kafkala et al. [35], the metals Co and Ni in Lavreotiki are of geogenic origin, and their elevated levels are not due to anthropogenic pollution. Nickel, although elevated in the studied soils (by 2.8 and 1.9 times at sites A and B), is an essential micronutrient required in small quantities for key physiological processes, including nitrogen metabolism. However, like other micronutrients such as Cr and Cu, it can become phytotoxic when present at elevated concentrations. In the studied sites, total soil concentrations of Cr and Cu exceeded threshold or background levels by up to approximately 3.5 and 5 times, respectively (Table 2). Despite these exceedances, the concentrations of Ni, Cr, and Cu observed are not considered high enough to pose significant environmental or phytotoxic risks under current conditions, particularly given the apparent tolerance of native plant species.

In contrast, the concentrations of Cd, Zn, Pb, Sb, and especially As in the studied soils were extremely elevated, far exceeding established threshold or background levels. Based on total concentration data, Cd levels were up to 138 times higher, Zn up to 64 times, and Pb up to 90 times the regulatory limits. The most extreme enrichments were observed for Sb and As, with Sb reaching up to 370 times and As reaching up to 686 times above background levels, depending on the site and reference value. Site-specific patterns were also evident: Ni and Zn concentrations were notably higher at the mining site (site A), while As, Cu, and Mn were elevated at the metallurgical site (site B), a likely reflection of differences in historical processing and waste deposition. Despite variations, both sites showed consistently high contamination with Cd, Pb, and Sb, indicating shared pollution sources and long-term accumulation in the substrate.

Importantly, the simultaneous elevation of multiple toxic elements—including As, Cd, Pb, Sb, and Zn—raises concerns about cumulative or synergistic effects on soil organisms and plant health, which may be more severe than the effects of individual elements in isolation. This highlights a key strength of the present study: its comprehensive, multi-element assessment of PTE contamination under realistic field conditions. While these concentrations significantly exceed typical ecotoxicological thresholds and pose a risk of chronic exposure, the continued presence of diverse native plant communities in the area suggests that many species have developed mechanisms of adaptation and metal tolerance, enabling their survival and establishment under prolonged PTE stress.

The total concentrations of elements in the soil do not necessarily correlate directly with their mobility, availability, or potential toxicity to organisms living there, including plants. The phytoavailability of metal elements in soil is influenced by several factors, including soil pH, organic matter content, texture, cation exchange capacity, the cumulative effect, and the presence of competing ions. Soil texture and microbial activity also affect metal retention, with clay soils generally retaining more metals than sandy soils. Furthermore, different plant species have varying capacities for metal uptake, which are influenced by root exudates and environmental conditions [34,36]. In fact, there are several standards and methods for assessing metal “availability” in soils [37]. Using different extractants and protocols can provide different results, therefore, their interpretation should be performed with a caution.

Due to the high pH values of the studied soils (7.58–7.62), the pool of elements in the soil solution–and thus potentially accessible for plants—can be expected to be relatively low. Indeed, the data presented in Table 2 reveal that the DTPA-extractable fractions of all studied elements, except for Cd, were markedly lower than their total concentrations (at the level 0.5–4.6% of their total concentrations), indicating a lower bioavailability of PTEs relative to their total content. In contrast, Cd exhibited notably higher DTPA-extractable concentrations (21–27%), indicating greater bioavailability, particularly in the rhizosphere where the pH is typically lower. However, despite elevated total concentrations, the DTPA-extractable fractions of Cd and other elements remained relatively low, suggesting both a potential risk of mobilization and a potential buffering capacity within the soil matrix.

### 2.2. Plant Diversity and Floristic Analysis

Despite the elevated soil pollution at the study sites, a relatively high degree of plant species richness was observed. The floristic survey conducted across the mining and metallurgical sites enabled the establishment of a comprehensive plant checklist, available in Table 3.

**Figure 1:**
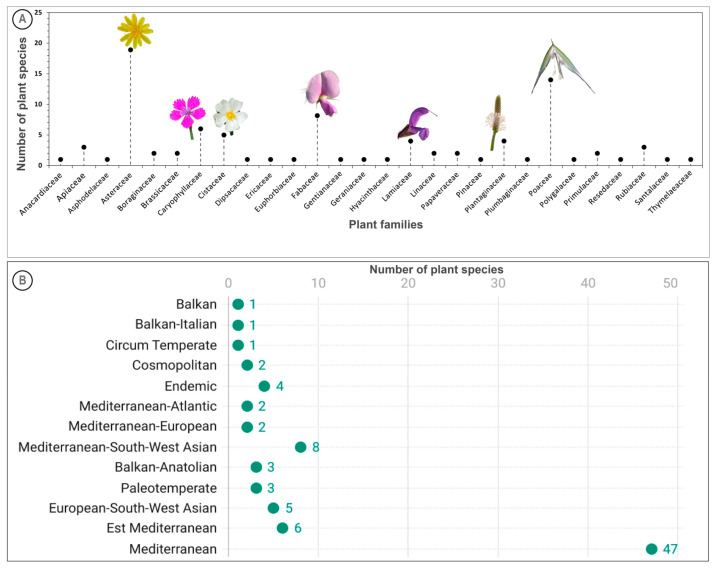
Comprehensive floristic analysis of native flora. (**A**) Diversity of taxonomical families; (**B**) chorology of plant species.

In total, 28 plant families were represented. The dominating plant families colonizing the study sites are illustrated in Figure 1. Asteraceae was the most species-rich family, comprising 19 species, followed by Poaceae (14 species) and Fabaceae (9 species). These families are also known to constitute a significant portion of the Mediterranean flora [38,39,40]. A notable feature of the floristic composition in this study is that 50% of the total number of plant families recorded at both sites were represented by only a single species, representing almost 15% of all species identified. This is a common characteristic of the flora of mining areas [41], suggesting multiple origins of PTEs tolerance in plants.

The study area is located within the Sterea Ellas (StE) floristic region, which belongs to the hotspot of Central and South Greece, one of the ten recognized hotspots of plant endemism and species richness in the Mediterranean Basin [42]. This region harbors numerous endemic species and serves as a crucial transitional zone between northern and southern Greece. Among the 89 native vascular plant taxa (species and subspecies) inventoried, nine taxa, namely, *Asperula lutea* subsp. *rigidula*, *Centaurea laureotica*, *Centaurea raphanina* subsp*. mixta*, *Crepis neglecta* subsp. *graeca*, *Dianthus serratifolius* subsp. *serratifolius*, *Erysimum graecum*, *Onobrychis ebenoides*, *Scorzonera crocifolia*, and *Silene corinthiaca*, are considered endemic to Greece. *Centaurea laureotica* is a local endemic of the Lavreotiki Peninsula.

Analysis of the recorded plant species distribution reveals that the majority, comprising 47 species, originate from the Mediterranean region (Figure 1B). Additionally, a smaller group of eight species is linked to Mediterranean-South-West Asian lineages. Plants of Mediterranean origin are usually well-adapted to harsh, arid environments and frequent human disturbances, making them effective colonizers of mining sites. These plants exhibit remarkable adaptations to hot, dry climates, and low soil fertility, allowing them not only to survive but often to dominate in these harsh environments [43,44].

**Figure 2:**
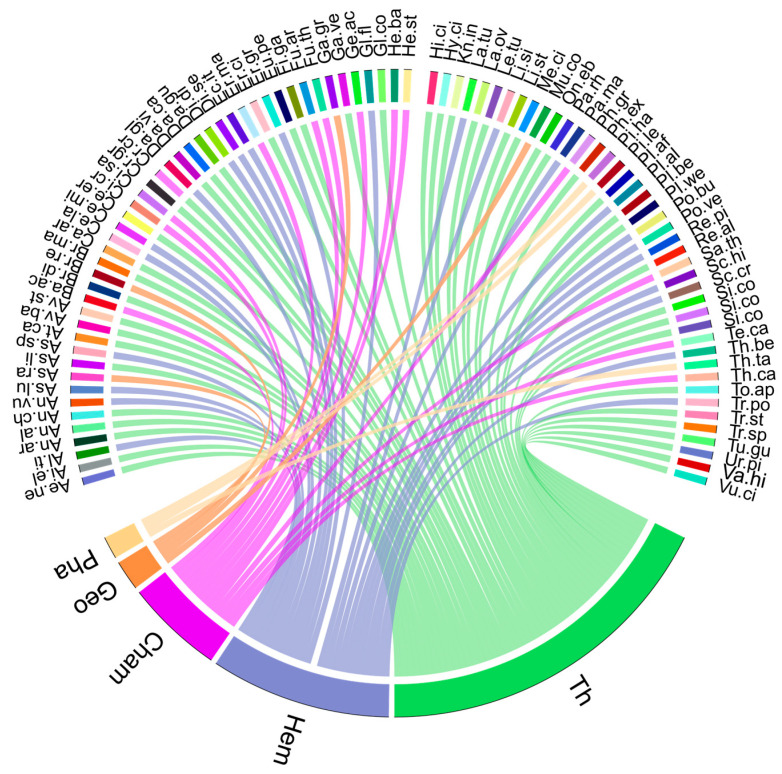
Comprehensive floristic analysis of native flora—distribution of life forms within the flora; Th—therophytes, Hem—hemicryptophytes, Cham—chamaephytes, Geo—geophytes, Pha—phanerophytes. For abbreviations of plant species see Table 3.

The inventory revealed a diverse spectrum of life forms with varying abundances, as shown in Figure 2. Therophytes were dominant, comprising 47 taxa, and accounting for 52.22% of the total flora. This predominance reflects the semi-arid Mediterranean bioclimate of the study area. In addition to therophytes, hemicryptophytes were significantly represented, constituting 26.67% of the surveyed flora. Chamaephytes showed a moderate presence at 13.33%, while geophytes and phanerophytes were the least occurring, representing 4.44% and 3.33%, respectively. The dominance of the Asteraceae family and the therophytic life form is attributed to their adaptations to ecologically disturbed zones [45]. It is well-documented that taxa within the Asteraceae family possess reproductive strategies that enhance their fitness and colonization potential [46]. For instance, many Asteraceae species produce seeds with a pappus—a plumose appendage—that facilitates wind-assisted seed dispersal, significantly increasing their colonization range [47]. Additionally, Asteraceae exhibit polymorphisms in seed and fruit morphologies, enabling diverse germination strategies that are advantageous in disturbed habitats. Similarly, the Poaceae family, which holds the second position in species richness, is renowned for its resilience in adverse conditions and its effective anemochorous (wind-assisted) seed dispersal strategies [48].

### 2.3. PTE Concentrations in Plant Aerial Parts

PTE concentrations were assessed in the aerial parts of 28 dominant plant species spontaneously colonizing the polluted areas at site A (13 taxa) and site B (15 taxa), including five species common to both locations (Table 4). Among the analyzed elements, both magnesium (Mg) and manganese (Mn) showed consistently low concentrations in plant tissues, particularly at site B, and generally fell within the ranges typical for healthy leaves of non-contaminated species [34]. The slightly alkaline pH and the presence of marble debris in the parent material suggest that the soils are carbonate-rich. This geochemical context likely contributes to the reduced bioavailability of Mg and Mn through ionic competition, precipitation, and retention in insoluble forms.

Despite elevated total Mn concentrations in the soils, especially at site B, Mn availability appeared limited, as indicated by low DTPA-extractable fractions (Table 2) and correspondingly low Mn accumulation in plant tissues. This is consistent with the reduced solubility of Mn at an alkaline pH and may be further exacerbated by competitive uptake inhibition from co-occurring metals such as Zn, Fe, and Pb. These factors, combined with potential exclusion mechanisms, suggest that native plants may actively restrict Mn translocation to aerial parts as an adaptive response to metal stress.

Similarly, Mg concentrations in plant tissues were low across multiple species. This may result from ionic competition with calcium and potassium, common in carbonate-rich soils, as well as from metal-induced disruptions of nutrient homeostasis. Additionally, some species may exhibit inherent physiological mechanisms that limit Mg uptake or translocation under conditions of heavy metal exposure. These findings emphasize that nutrient acquisition in metalliferous soils is governed not only by total element content, but also by bioavailability, ion interactions, and species-specific adaptive strategies.

Several species, particularly those growing at site A (the former mining area), exhibited elevated concentrations of multiple potentially toxic elements (PTEs), including As, Cd, Cr, Fe, Ni, Pb, Sb, and Zn. These elevated values showed considerable variability across species, reflecting differences in metal accumulation strategies, tolerance mechanisms, and species-specific physiological traits. Notably, many of the measured concentrations exceeded the typical background levels reported for naturally grown, non-contaminated plants [34], suggesting that these species are either capable of tolerating high internal metal loads or are selectively accumulating specific elements.

At site A, the highest concentrations of specific elements were recorded in distinct plant species, highlighting notable interspecific variation in metal accumulation. *Helichrysum stoechas* accumulated the greatest levels of As (6.24 mg kg^−1^) and Pb (463.60 mg kg^−1^), both of which significantly exceed typical background levels for non-contaminated plants. *Phagnalon graecum* showed elevated concentrations of Cd (25.54 mg kg^−1^) and Cu (7.06 mg kg^−1^), while *Brachypodium retusum* had the highest values for Co (1.08 mg kg^−1^), Ni (4.04 mg kg^−1^), and Cr (5.64 mg kg^−1^), indicating a potential for co-accumulation of transition metals. *Scorzonera crocifolia* accumulated the most Mg (2386.17 mg kg^−1^), within the upper range of normal levels, and *Cistus salviifolius* and *Dianthus diffusus* exhibited the highest concentrations of Mn (30.31 mg kg^−1^) and Zn (619.66 mg kg^−1^), respectively. Several of these concentrations, particularly for As, Cd, Pb, and Zn, substantially exceeded the typical values reported for uncontaminated plant tissues (Table 4), suggesting potential for metal tolerance or selective accumulation mechanisms.

At site B, *Alkanna tinctoria* consistently exhibited the highest concentrations across all analyzed elements, including As (31.02 mg kg^−1^), Cd (4.37 mg kg^−1^), Co (0.27 mg kg^−1^), Cr (2.86 mg kg^−1^), Cu (11.22 mg kg^−1^), Fe (930.81 mg kg^−1^), Mg (1738.68 mg kg^−1^), Mn (52.72 mg kg^−1^), Ni (3.08 mg kg^−1^), Pb (304.89 mg kg^−1^), Sb (6.75 mg kg^−1^), and Zn (336.88 mg kg^−1^). While concentrations of elements such as Co, Cr, Cu, Mg, and Mn remained within or only slightly above normal physiological ranges, levels of As, Cd, Pb, and Sb were significantly elevated.

Notably, the extremely high soil arsenic concentrations at site B (3184.87–3430.68 mg kg^−1^; Table 2) were reflected in the elevated As levels detected across all dominant plant species at the site, suggesting that despite the low bioavailability typically associated with alkaline conditions, some degree of arsenic uptake still occurred—likely facilitated by species-specific physiological adaptations or localized rhizosphere processes enhancing As solubility.

**Figure 3:**
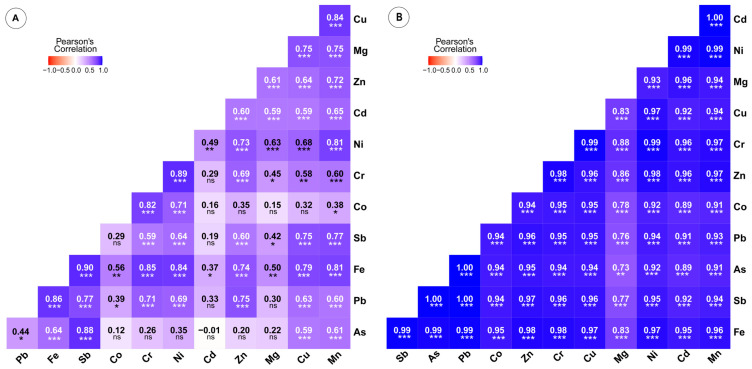
Correlation coefficient heatmap for potentially toxic element concentrations at sites (**A**) and (**B**). ns = non-significant; significant at: * *p* < 0.05; ** *p*< 0.01; and *** *p* < 0.001.

The uptake of PTEs by plants from the soil is influenced by the plant species, the total concentrations of the elements and their availability, the soil pH, the physiology of the plant, and its maturity [49]. At site A, plant species such as *Brachypodium retusum*, *Dianthus diffusus*, and *Knautia integrifolia* display higher concentrations of Cd, Cr, Cu, Pb, and Zn compared to site B, where *Alkanna tinctoria* exhibits notable levels of certain elements. Overall, plants from site A show higher element concentrations across most species. Variability in concentrations of PTEs such as Cd, Pb, and Ni is observed among different plant species at both sites. Analyzing the accumulation potential of the plant species, *Asperula lutea* subsp. *rigidula* at site A and *Alkanna tinctoria* at site B stand out for their high concentrations of multiple elements. Conversely, species like *Thymbra capitata* and *Brachypodium retusum* consistently show low element concentrations at both sites. Furthermore, investigations into element correlation and co-occurrence reveal potential associations between certain elements within plant species, suggesting potential co-accumulation patterns or shared uptake mechanisms. Understanding these correlations can provide insights into the elemental relationships within plant tissues (Figure 3).

### 2.4. Bioconcentration Factor

The bioconcentration factor (BCF) is the ratio of the element concentration in the plant aerial parts to its concentration in the soil [50]. It provides important information about plant capacity to take up an element from the soil and store it in the aerial parts. Therefore, this parameter is often used to estimate the metalloid accumulation strategy of plants and thus their usefulness for various phytoremediation practices [50,51,52]. In theory, plants with BCF > 1 are able to accumulate PTEs in their aerial parts and can eventually be candidates for phytoextraction. On the other hand, plants with BCF < 1 are considered as excluders and have more potential for phytostabilization.

The BCF across various plant species at the two polluted sites were evaluated (Table 5). Plant species at site A exhibited higher BCF compared to those at site B. The variation in BCF among different plant species was substantial at site A, whereas differences remained statistically insignificant at site B.

It is often considered that contrasting BCFs between plant species reflect their different resilience to metal concentrations in the soil [53]. However, interpretation of BCF in this context should be performed with caution [54]. It has been suggested that the level of plant tolerance to PTEs should rather be determined by BCF based on the extractable (potentially bioavailable) pool of elements in the soil with such BCF > 1 combined with lack of toxicity symptoms indicating high tolerance [55]. In many studies, including ours, this cannot be applied since the extractable pools of many elements were very low or even below the detection limits (Table 4). Among the plant species collected, the highest BCF value for As was observed in *Alkanna tinctoria* at site B. For Cd, the highest BCF values were recorded for *Phagnalon graecum* and *Scorzonera crocifolia*, all originating from site A. Regarding Cr, the greatest BCF value appeared at site B in *A. tinctoria*. BCF for Cu ranged from 0.0003 to 0.0419, with the highest value recorded in *P. graecum* at site A. The highest values for Ni occurred in *A. tinctoria* (0.0209 ± 0.0) at site B and *Brachypodium retusum* (0.0201 ± 0.006) at site A. Lead exhibited the highest BCF in *Helichrysum stoechas* subsp*. barrelieri* and *A. tinctoria* at sites A and B, respectively. BCF values for Sb were very low, with the highest recorded in *A. tinctoria* at site B. The BCF for zinc displayed its peak values in *Dianthus diffusus*, *Asperula lutea* subsp. *rigidula*, and *H. stoechas* subsp*. barrelieri*. Remarkably, all BCF values were consistently below 1. Similar observations were seen for other plant species colonizing spontaneously extremely metal-polluted habitats around the world, for instance in Poland [54], Spain [56], or Morocco [57]. Therefore, the native plant species of the studied metalliferous sites can be considered as candidates for phytostabilization practices.

### 2.5. Candidate Plant Species for Phytostabilization of Polluted Areas

Many of the semi-arid Mediterranean flora studied in this work, including fast-growing therophytes and chamaephytes (Table 3 and Figure 2), follow an r-strategy of rapid reproduction, often forming dense ground cover that stabilizes soil and traps contaminants [58,59,60,61]. This vegetation acts as a seed bank and improves conditions for other species via “nurse plant” effects [62]. By cooling the microenvironment and enhancing soil quality, these plants support phytostabilization and revegetation [63,64].

**Figure 4:**
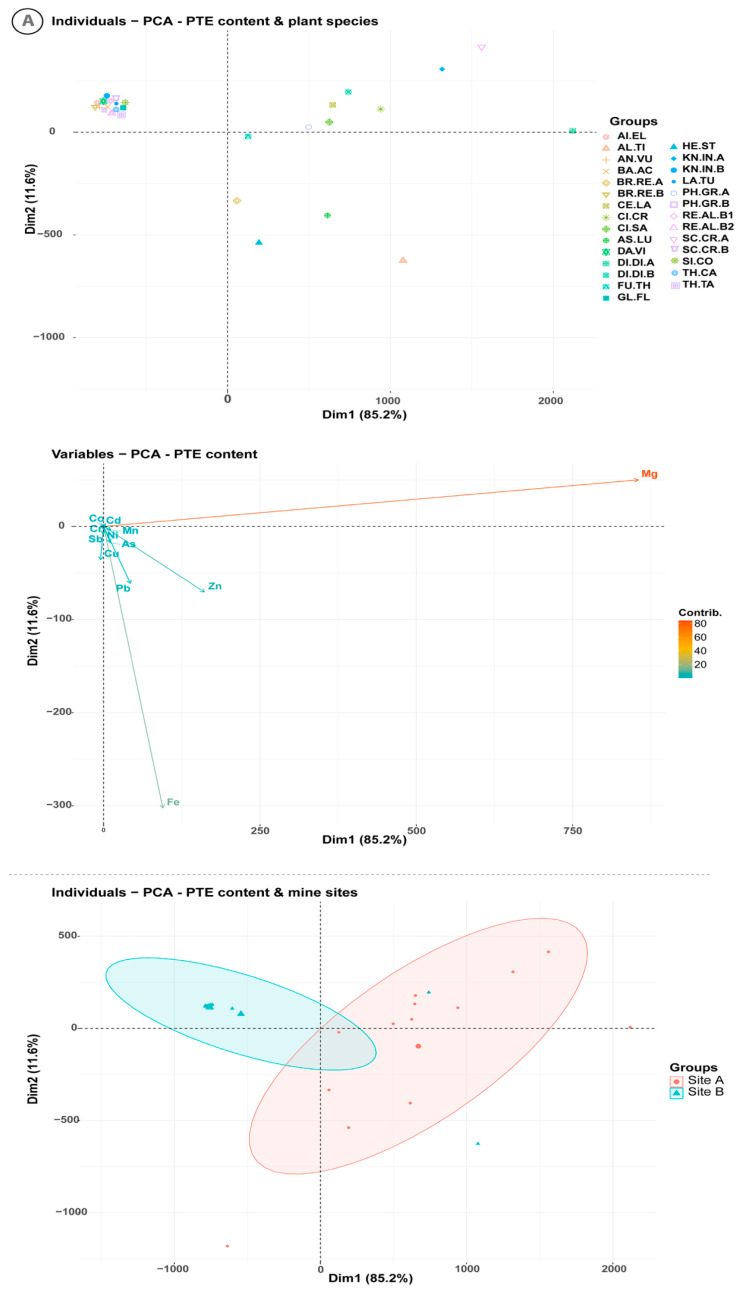

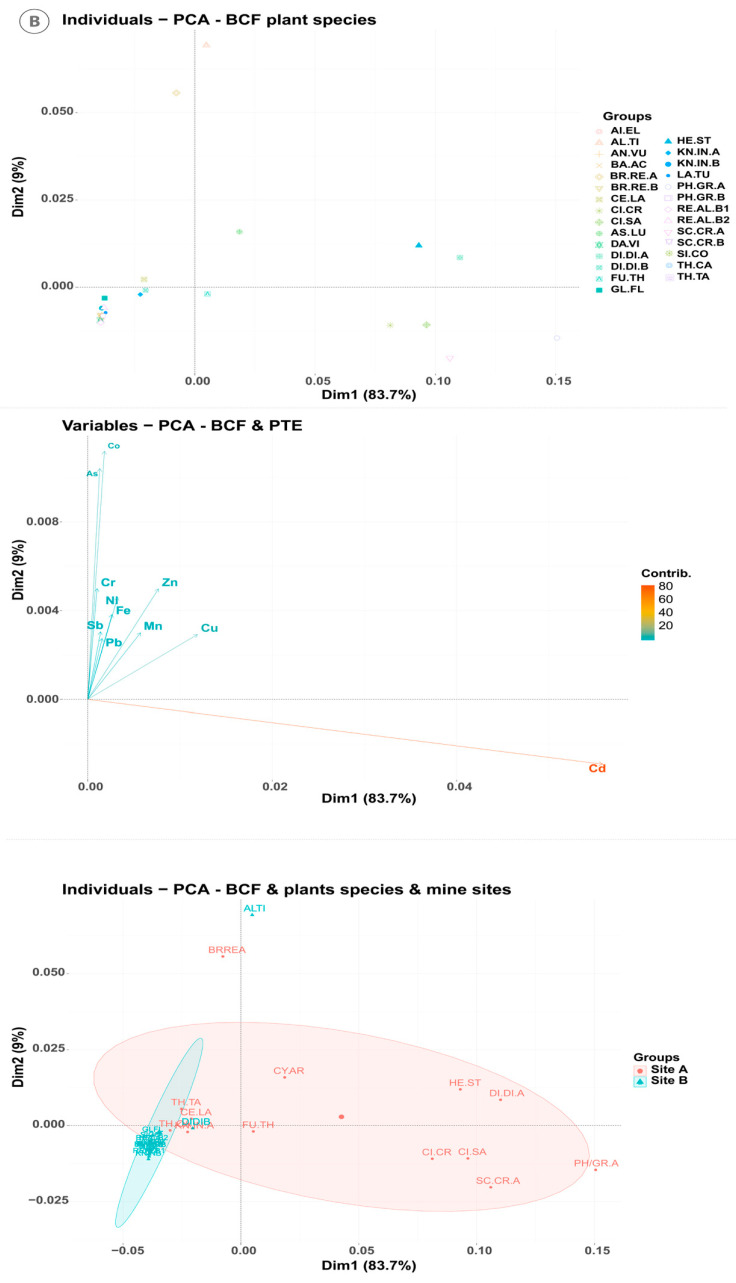
(**A**) Principal component analysis (PCA) of the concentrations of the studied potential toxic elements in plant aerial parts at mining site A and metallurgical site B. (**B**) Principal component analysis (PCA) of the bioconcentration factors (BCF) of the studied potential toxic elements in plant aerial parts at mining site A and metallurgical site B.

The principal component analysis (PCA) biplot, derived from PTE concentrations in aerial plant parts, explained 96.8% of the total variance (Figure 4A). The first principal component (Dim1) accounts for 88.73% of the variance, elucidating the capacity of the native plant species to simultaneously accumulate diverse PTEs. This component distinctly identifies *Asperula lutea* subsp. *rigidula* for its elevated concentrations of Zn in its aboveground parts. Additionally, Dim1 underscores the high concentrations of Cu, Pb, Sb, and As in *Brachypodium retusum*, *Helichrysum stoechas* subsp. *barrelieri*, as well as Ni in *Thymelaea tartonraira*, and *Cistus salviifolius*.

The second principal component (Dim2), which accounts for 11.6% of the total inertia, represents the concentration gradients of As, Cd, Co, Cr, Cu, Fe, Mg, Mn, Ni, Pb, Sb, and Zn. This component highlights a notable affinity of *Fumana thymifolia* for Ni and As, as well as of *Alkanna tinctoria* and *Asperula lutea* subsp. *rigidula* for Zn, and *Brachypodium retusum* and *Helichrysum stoechas* subsp. *barrelieri* for Cu, Pb, Sb, and As. On the other hand, the lowest metal accumulation was found in *Lactuca tuberosa* at site A, *Anthyllis vulneraria*, *Dasypyrum villosum*, *Alkana tinctoria*, *Silene corinthiaca*, *Glaucium flavum* at site B, and *Aira elegantissima* in both sites. The size of the ellipses around each group indicates the variability within the group, with a larger ellipse for site A suggesting greater diversity within that group compared to site B. This observation reflects the disparity in the concentrations of the PTEs studied. 

The biplot resulting from the PCA on the BCF and PTEs in the soil accounts for 92.7% of the total variance in the data (Figure 4B). The first principal component (Dim1) explains 83.7% of the total inertia, while the second principal component (Dim2) provides additional insight, accounting for 9% of the total inertia. The PCA analysis confirms, that the species characterized by the highest BCF are *Helichrysum stoechas*, *Cistus salviifolius*, *Cistus creticus*, *Asperula lutea* subsp. *rigidula*, *Fumana thymifolia* at site A, and *Dianthus diffusus* in both sites, whilst the lowest BCF is found in *Knautia integrifolia* at site A, *Glaucium flavum*, *Reseda alba*, *Knautia integrifolia*, *Ballota acetabulosa* and *Dasypyrum villosum* at site B. The data form two distinct clusters (Figure 4B): site A, characterized by huge diversity of the species in terms of BCF, and site B, where the species show a relatively narrow range of BCF variability.

Using native species that naturally colonize metalliferous areas can enhance phytoremediation success [65]. These plants are adapted to local conditions and require minimal maintenance, making them ideal candidates for ecological restoration [41]. Effective phytostabilizers typically exhibit low metal translocation, dense growth, robust root systems, and high PTE tolerance. Based on these criteria, species such as *Cistus creticus*, *Cistus salviifolius*, *Thymelaea tartonraira*, *Ballota acetabulosa*, *Fumana thymifolia*, and *Helichrysum stoechas* subsp*. barrelieri* are strong candidates for restoring contaminated Mediterranean mining and metallurgical sites.

## 3. Materials and Methods

### 3.1. Study Sites Description

This study was conducted at two sites on the Lavreotiki Peninsula in the Attica Prefecture, Greece, a region well-known for its historical mining and metallurgical activities dated back to 3500 BC. The region was historically exploited for argentiferous galena, a lead ore rich in silver, which played a pivotal role in supporting the economic foundation of ancient Athens—particularly through the minting of the Athenian drachma. Historical accounts estimate that around 3500 tons of silver and 1.4 million tons of lead were extracted from this area, with peak production occurring during the 5th and 4th centuries BC, when nearly 70% of total output was generated [27]. Mining operations declined by the 3rd century BC and ceased entirely by the 1st century BC. Industrial-scale mining and smelting resumed in the mid-19th century and persisted into the 1980s, reaffirming Lavreotiki’s role as a major metallurgical hub. By the turn of the 20th century, Lavrion smelters accounted for approximately 3% of global lead production [27]. This extensive history of ore extraction and processing has resulted in widespread distribution of potentially toxic elements (PTEs) across urban, peri-urban, and natural landscapes in the Lavrion area, creating a legacy of contamination that now serves as a valuable context for studying plant adaptation and site-specific phytoremediation potential.

**Figure 5:**
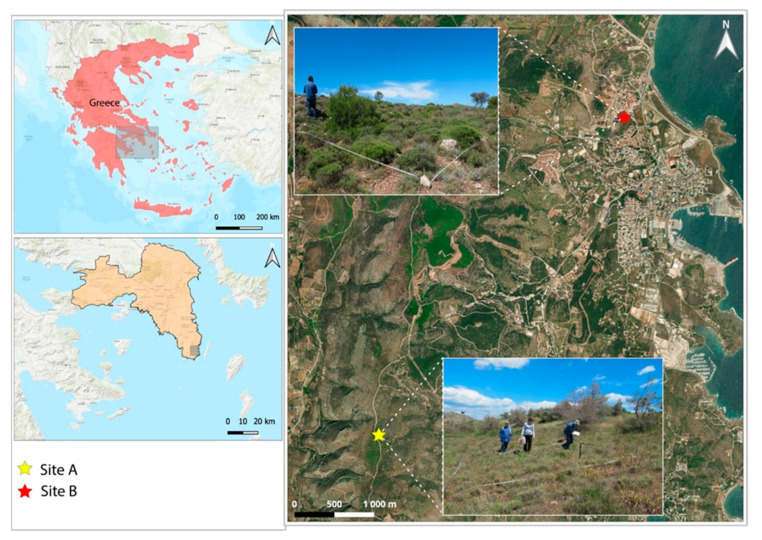
Localization of the studied sites.

The selected sites were the Michaly mining site (site A: 37°41′15.50″ N, 24°0′52.41″ E) and the Varvara metallurgical site (site B: 37°43′23.6″ N, 24°02′58.6″ E) (Figure 5). These two sites were selected based on prior investigations and soil analyses, which identified these sites as being heavily contaminated by PTEs. Both locations also exhibit spontaneous colonization by native plant communities, making them well-suited for evaluating plant responses to long-term contamination in different types of industrially impacted environments.

The Michaly site, situated in the Sounio region, was used for metal mining and waste disposal. The topography of this area is characterized as a hillside with a 15% slope, a northern orientation, and an altitude of approximately 220 m. The primary geological substrate is mica schist, with a stone and gravel content of around 10%, including marble debris. The Varvara site is located near the center of the city of Lavrio, approximately 60 km southeast of Athens. The topography of the area is characterized as a hillside with a 10% slope, a north-western orientation, and an altitude of about 45 m. The primary geological substrate consists of schist (phyllitic cover), with a stone and gravel content of less than 10%. The distance between the two sites is 8.8 km.

Both sites are in sparse, degraded *Pinus halepensis* Mill. woodlands dominated by dwarf, cushion-shaped thorny shrubs (phrygana). These formations typically support high plant species richness, with a significant component of annual plants.

According to the Köppen–Geiger climatic classification, the study area belongs to the Csa (hot-summer Mediterranean climate) class [66] with an annual precipitation of 400–450 mm and a mean annual temperature of 16.5 to 18.5 °C. The bioclimate of this region is characterized as dry by the De Martonne aridity index [67] and semi-arid by the Emberger index [68].

At each site, three randomly selected square plots, each with a size of 5 × 5 m, were marked for soil analysis and floristic inventory.

### 3.2. Soil Sampling and Analyses

From each plot, three soil samples were collected at a depth of 0–30 cm, air-dried at room temperature, ground into a fine powder, and sieved through a 2.0 mm mesh. Soil texture was determined using the sieving and pipette method [69]. Soil pH was determined in 1:1 soil/distilled water suspension after 1 h with a HI 2211 pH meter (Hanna Instruments, Padova, Italy). The organic matter content was analyzed by the Walkley–Black method [70]. Soil conductivity was measured with a WTW LF 537 electrode (Wissenschaftlich-Technische Werkstätten, Weilheim, Germany) after a 30 min equilibration in deionized water at a liquid-to-solid ratio of 5:1, followed by filtration with Schleicher and Schuell white ribbon filter paper (Dassel, Germany) [71]. The cation exchange capacity (CEC) was determined by saturating the soil matrix with NH_4_^+^, desorbing it with K^+^ and quantifying the NH_4_^+^ in the leachate [72].

To evaluate the total concentrations of As, Cd, Co, Cr, Cu, Fe, Mn, Ni, Pb, Sb, and Zn, 500 mg of air-dried soil was digested in 4 mL of aqua regia (a mixture of HNO_3_ and HCl in a 1:3 ratio) for 30 min in a microwave oven (Milestone, 1200 MEGA, Gemini, The Netherlands). In addition, the diethylenetriaminepentaacetic acid (DTPA)-based extraction [73] was performed by mixing 10 g of ground soil with 20 mL of DTPA (Sigma-Aldrich, Burlington, MA, USA) solution (0.005 M DTPA, adjusted to pH 7.3). DTPA extraction is widely used to estimate the bioavailable fraction of metals in soils, particularly in moderately to slightly alkaline conditions, such as those found in the Lavreotiki Peninsula. The elemental concentrations in the extracts were quantified using inductively coupled plasma optical emission spectrometry (ICP-OES, Agilent Technologies 700 Series, Santa Clara, CA, USA). The CRM143R sewage sludge amended soil was used as a standard reference material (SRM).

### 3.3. Plant Collection and Identification

The same plots were used to study the floristic composition of the two polluted sites, A and B. All plant species growing within the plots were recorded at the peak of the flowering season (April 2019). Specimens from all recorded species were collected and identified using identification keys [74,75,76]. Plant nomenclature follows [38]. Voucher specimens were deposited at the Herbarium of the Agricultural University of Athens (AUA). Aerial parts (including leaves, stems, and inflorescence) from all dominant plant species at the study sites were collected, air-dried, and stored for further analyses. For each species, a minimum of three specimens were randomly collected within each sampling plot. Plant samples intended for species identification were kept in a plant press and separated from those designated for analysis.

### 3.4. Potential Toxic Elements Concentration and Bioconcentration Assessment

The dominant plant species present at both sites were analyzed for their PTEs accumulation and bioconcentration properties. Plant aerial parts (leaves, stems, and inflorescence) were analyzed for the same elements as for soils, with the addition of Mg. Magnesium was included in the plant analysis due to its physiological importance as an essential macronutrient and to assess whether its uptake might be influenced by the presence of high concentrations of PTEs. Magnesium was not analyzed in soils, as it is not classified as a potentially toxic element and is not typically associated with mining-related contamination.

The aerial parts of each plant were thoroughly washed under running tap water, followed by a 15 s wash with a phosphate-free detergent solution, and another 15 s rinse with tap water. Finally, the samples were carefully rinsed twice with deionized water [77], and oven-dried until a constant weight was achieved. To determine the total PTEs concentrations in the plant tissues, the dried plant material was hammer-milled (SM100, Retsch GmbH, Germany) to obtain a fine powder and then wet-digested in Pyrex tubes using a heating block. Specifically, the digestion process involved three cycles with 1 mL HNO_3_ (70%) and one cycle with 1 mL HCl (37%) at 120 °C for 4 h. After digestion, the samples were dissolved in HCl (37%) and diluted to a final volume of 5 mL (2% HCl) using Millipore water. The resulting extracts were analyzed using an ICP-OES (Agilent Technologies 700 Series, Santa Clara, CA, USA). All samples were analyzed in triplicate. For quality control, a blank and a standard reference material (NIST SRM 1570a, Spinach leaves) [78]) were included in the analysis.

The phytoaccumulation efficiency of the selected native plants, as well as their capacity to accumulate trace elements in their aboveground parts, were evaluated by calculating the bioconcentration factor (BCF) using the following equation:

BCF = [PTE]aboveground plant parts/[PTE]soil

where [PTE] is the concentration of PTE.

### 3.5. Statistical Analysis

Statistical differences in the concentrations of As, Cd, Co, Cr, Cu, Fe, Mg, Mn, Ni, Pb, Sb, and Zn in the soil at sites A and B, as well as among different plant species, were evaluated using a one-way analysis of variance (ANOVA), followed by Tukey’s post hoc test to determine significant differences between sites and plant species. All statistical analyses were performed using SPSS Statistics version 21 (IBM Corp., Armonk, NY, USA), with *p*-values ≤ 0.05 considered statistically significant. Additionally, a principal component analysis (PCA) was conducted using R 4.0.3 software (R Core Team, 2022) to visualize the concentrations of the PTEs in the studied plant species.

## 4. Conclusions

This study investigated native Mediterranean plant species that have naturally colonized soils historically contaminated by mining and metallurgical activities. By examining these spontaneously established communities, the study aimed to identify species capable of tolerating high concentrations of potentially toxic elements (PTEs) and to evaluate their suitability for phytoremediation and ecological restoration. High concentrations of As, Cd, Cu, Mn, Pb, Sb, and Zn were detected in the soils of the study sites at Michaly and Varvara, at the Lavreotiki peninsula, Greece. Despite these environmental constraints, the floristic inventory revealed high plant species richness, dominated by therophytes, consistent with the arid bioclimatic conditions of the region. The elemental analysis of plant tissues showed that although several species accumulated elevated levels of certain elements in their aerial parts, none exhibited bioaccumulation factors above 1 for PTEs, indicating limited capacity for active accumulation. These findings suggest that none of the studied species are suitable for phytoextraction, primarily due to their limited aboveground biomass and growth characteristics. However, several species exhibited low metal accumulation in aerial tissues (BCF < 1) and clear tolerance to high concentrations of potentially toxic elements in the soil. In addition, their morphological traits (such as being perennial, low-growing, or forming dense ground cover) are favorable for minimizing soil erosion and stabilizing contaminated surfaces, indicating their potential suitability for phytostabilization strategies. Based on these criteria and their spontaneous establishment in contaminated soils, species such as *Cistus creticus*, *Cistus salviifolius*, *Thymelaea tartonraira*, *Ballota acetabulosa*, *Fumana thymifolia*, and *Helichrysum stoechas* subsp*. barrelieri* emerged as promising candidates for phytostabilization and ecological restoration of degraded Mediterranean landscapes. Future phytomanagement strategies in arid and semi-arid environments will benefit from a deeper understanding of native plant community structures, soil–plant biogeochemical interactions, and ecosystem functional traits. In particular, the roles of rhizospheric microbes and fungi, known to enhance plant performance under stress, deserve closer investigation. Likewise, advances in plant ecophysiological research, including metabolomic, proteomic, transcriptomic, and genomic approaches, could further inform selection and enhancement of phytomanagement species. Overall, this study meets its objectives and highlights the importance of long-term contaminated areas as “living laboratories,” where native flora, some of which are endemic to Greece and the Lavreotiki Peninsula, have adapted over centuries to metalliferous substrates. These landscapes not only offer ecological value for restoration but also warrant protection and conservation due to their unique biodiversity and scientific potential.

## Figures and Tables

**Table 1:** Soil physicochemical characteristics.

	Site A	Site B
Clay (%)	8.0	17.0
Silt (%)	21.0	18.0
Sand (%)	71.0	65.0
Texture	Sandy loam	Sandy loam
pH	7.62	7.58
Organic matter (g 100^−1^ g DW)	4.4	9.6
Conductivity (μS/cm)	225	300
CEC (cmol(+)kg^−1^, DW)	18	18

**Table 2:** Range of total and DTPA-extractable metal and metalloid concentrations (mg kg^−1^) in soils from sites A and B. Mean values were used for statistical analysis.

PTE	Site A (Mining Area)		Site B (Metallurgical Area)		Threshold Values (♦) & Natural Background Levels (‡) in Soil (mg kg^−1^)
	Total Concentrations	DTPA Extractable Concentrations	Total Concentrations	DTPA Extractable Concentrations	
As	387.67–497.08 ^a^	0.1–0.3 ^A^	3184.87–3430.68 ^b^	0.27–0.68 ^B^	5.0–6.0 ‡
Cd	132.05–138.05 ^a^	27.3–37.8 ^A^	128.86–132.45 ^a^	17.80–27.78 ^Β^	1.0–3.0 ♦
Co	8.32–13.11 ^a^	0.08–0.23 ^A^	5.23–7.56 ^b^	0.02–0.04 ^B^	20.0–50.0) ‡
Cr	198.92–356.80 ^a^	<D.L.	127.36–242.44 ^a^	<D.L.	10.0–100.0 ‡
Cu	144.00–168.45 ^a^	1.0–1.21 ^A^	601.67–724.91 ^b^	16.19–26.53 ^B^	50.0–140.0 ♦
Fe	28,597.99–39,175.34 ^a^	0.41–0.97 ^A^	61,058.76–62,947.18 ^b^	0.56–0.88 ^B^	10,000.0–100,000.0 ‡
Mn	1545.68–1797.30 ^a^	0.18–0.25 ^A^	2653.39–2948.21 ^b^	0.22–0.39 ^B^	350.0–2000.0 ‡
Ni	179.76–200.84 ^a^	1.15–2.10 ^A^	124.52–146.83 ^b^	1.45–2.47 ^B^	30.0–75.0 ♦
Pb	23,260.27–27,046.81 ^a^	323.23–425.15 ^A^	22,770.64–25,875.72 ^a^	119.14–132.31 ^B^	50.0–300.0 ♦
Sb	192.1–279.40 ^a^	0.07–0.29 ^A^	253.49–369.59 ^a^	0.30–0.62 ^B^	0.1–10.0 ‡
Zn	17,472.22–19,200.24 ^a^	665.81–751.55 ^A^	14,805.01–16,821.60 ^b^	741.50–788.72 ^B^	150.0–300.0 ♦

♦ Threshold values according to the European Soil Directive (86/278/EEC) [33]. ‡ Natural background levels according to Kabata-Pendias, 2011 [34]. <D.L. = below detection limit. Values followed by the same letters are not statistically different. Lowercase letters indicate significant differences in total metal concentrations, while uppercase letters indicate significant differences in DTPA-extractable concentrations.

**Table 3:** List of plant species recorded at the studied mining and metallurgical sites, along with their chorology and life form. Species in which potentially toxic elements were measured are indicated with an asterisk (*).

Plant Species	Family	Chorology	Life Form	Abbreviation	Location Site
*Aegilops neglecta*	Poaceae	Mediterranean-SW Asian	Therophyte	*Ae.ne*	B
Aira elegantissima *	Poaceae	Mediterranean-SW Asian	Therophyte	*Ai.el*	A, B
Alkanna tinctoria *	Boraginaceae	Mediterranean	Hemicryptophyte	*Al.ti*	B
*Anagallis arvensis*	Primulaceae	Cosmopolitan	Therophyte	*An.ar*	A
*Anthemis altissima*	Asteraceae	European-SW Asian	Therophyte	*An.al*	B
*Anthemis chia*	Asteraceae	Mediterranean	Therophyte	*An.ch*	B
Anthyllis vulneraria *	Fabaceae	European	Hemicryptophyte	*An.vu*	Β
*Asperula lutea* subsp. rigidula *	Rubiaceae	Greek endemic	Chamaephyte	*As.lu*	A
*Asphodelus ramosus*	Asphodelaceae	Mediterranean	Geophyte	*As.ra*	A, B
*Asterolinon linum-stellatum*	Primulaceae	Mediterranean	Therophyte	*As.li*	A
*Astragalus spruneri*	Fabaceae	Balkan	Hemicryptophyte	*As.sp*	A
*Atractylis cancellata*	Asteraceae	Mediterranean	Therophyte	*At.ca*	Β
*Avena barbata*	Poaceae	Mediterranean	Therophyte	*Av.ba*	Β
*Avena sterilis*	Poaceae	Mediterranean-SW Asian	Therophyte	*Av.st*	Β
Ballota acetabulosa *	Lamiaceae	Balkan-Anatolian	Chamaephyte	*Ba.ac*	Β
*Brachypodium distachyon*	Poaceae	Mediterranean-SW Asian	Therophyte	*Br.di*	A, B
Brachypodium retusum *	Poaceae	Mediterranean	Hemicryptophyte	*Br.re*	A, B
*Bromus madritensis*	Poaceae	Mediterranean-SW Asian	Therophyte	*Br.ma*	A, B
*Calendula arvensis*	Asteraceae	Mediterranean	Therophyte	*Ca.ar*	Β
Centaurea laureotica *	Asteraceae	Greek endemic	Hemicryptophyte	*Ce.la*	A
*Centaurea raphanina* subsp. *mixta*	Asteraceae	Greek endemic	Hemicryptophyte	*Ce.mi*	A, B
*Centaurium erythraea*	Gentianaceae	European-SW Asian	Therophyte	*Ce.er*	A, B
Cistus creticus *	Cistaceae	Mediterranean	Chamaephyte	*Ci.cr*	A
Cistus salviifolius *	Cistaceae	Mediterranean	Chamaephyte	*Ci.sa*	A
*Crepis neglecta* subsp. *graeca*	Asteraceae	Greek endemic	Therophyte	*Cr.gr*	A
*Crupina crupinastrum*	Asteraceae	European-SW Asian	Therophyte	*Cr.cr*	Β
*Dactylis glomerata*	Poaceae	Paleotemperate	Hemicryptophyte	*Da.gl*	A, Β
Dasypyrum villosum *	Poaceae	Mediterranean-SW Asian	Therophyte	*Da.vi*	Β
*Daucus carota*	Apiaceae	Paleotemperate	Therophyte	*Da.ca*	Β
*Daucus guttatus*	Apiaceae	Mediterranean	Therophyte	*Da.gu*	Β
Dianthus diffusus *	Caryophyllaceae	Balkan-Anatolian	Hemicryptophyte	*Di.di*	A, B
*Dianthus serratifolius* subsp. *serratifolius*	Caryophyllaceae	Greek endemic	Hemicryptophyte	*Di.se*	A, B
*Echium italicum*	Boraginaceae	Mediterranean-SW Asian	Hemicryptophyte	*Ec.it*	Β
*Erica manipuliflora*	Ericaceae	Mediterranean	Chamaephyte	*Er.ma*	A
*Erodium cicutarium*	Geraniaceae	Circumtemperate	Therophyte	*Er.ci*	Β
*Erysimum graecum*	Brassicaceae	Greek endemic	Hemicryptophyte	*Er.gr*	B
*Euphorbia peplus*	Euphorbiaceae	Cosmopolitan	Therophyte	*Eu.pe*	A, B
*Filago gallica*	Asteraceae	Mediterranean-Atlantic	Therophyte	*Fi.ga*	A
*Fumana arabica*	Cistaceae	Mediterranean	Chamaephyte	*Fu.ar*	A
Fumana thymifolia *	Cistaceae	Mediterranean	Chamaephyte	*Fu.th*	A
*Gagea graeca*	Liliaceae	Balkan-Anatolian	Geophyte	*Ga.gr*	A
*Gastridium ventricosum*	Poaceae	Mediterranean	Therophyte	*Ga.ve*	B
*Genista acanthoclada*	Fabaceae	Mediterranean	Chamaephyte, Phanerophyte	*Ge.ac*	A
Glaucium flavum *	Papaveraceae	Mediterranean-European	Hemicryptophyt	*Gl.fl*	B
*Glebionis coronaria*	Asteraceae	Mediterranean	Therophytes	*Gl.co*	B
*Helichrysum stoechas* subsp. barrelieri *	Asteraceae	Mediterranean	Chamaephyte	*He.st*	A
*Hippocrepis ciliata*	Fabaceae	Mediterranean	Therophyte	*Hi.ci*	Β
*Hymenocarpos circinnatus*	Fabaceae	Mediterranean	Therophyte	*Hy.ci*	Β
Knautia integrifolia *	Dipsacaceae	Mediterranean	Therophyte	*Kn.in*	A, B
Lactuca tuberosa *	Asteraceae	European-SW Asian	Hemicryptophyte	*La.tu*	A
*Lagurus ovatus*	Poaceae	Mediterranean	Therophyte	*La.ov*	Β
*Leotondon tuberosus*	Asteraceae	Mediterranean	Hemicryptophyte	*Le.tu*	A
*Limonium sinuatum*	Plumbaginaceae	Mediterranean	Hemicryptophyte	*Li.si*	Β
*Linum strictum*	Linaceae	Mediterranean	Therophyte	*Li.st*	A, B
*Medicago ciliaris*	Fabaceae	Mediterranean	Therophyte	*Me.ci*	B
*Muscari commutatum*	Hyacinthaceae	Balkan-Italian	Geophyte	*Mu.co*	Β
*Onobrychis ebenoides*	Fabaceae	Greek endemic	Hemicryptophyte	*On.eb*	A
*Papaver rhoeas*	Papaveraceae	Mediterranean	Therophyte	*Pa.rh*	Β
*Paronychia macrosepala*	Caryophyllaceae	Mediterranean	Hemicryptophyte	*Pa.ma*	A, Β
*Phagnalon rupestre* subsp. graecum *	Asteraceae	Mediterranean	Chamaephyte	*Ph.gr*	A, B
*Phleum exaratum*	Poaceae	Mediterranean	Therophyte	*Ph.ex*	B
*Pinus halepensis*	Pinaceae	Mediterranean	Phanerophyte	*Pi.ha*	A, B
*Pistacia lentiscus*	Anacardiaceae	Mediterranean	Phanerophyte	*Pi.le*	A, B
*Plantago afra*	Plantaginaceae	Mediterranean	Therophyte	*Pl.af*	Β
*Plantago albicans*	Plantaginaceae	Mediterranean	Hemicryptophyte	*Pl.al*	Β
*Plantago bellardii*	Plantaginaceae	Mediterranean	Therophyte	*Pl.be*	A, B
*Plantago weldenii*	Plantaginaceae	Mediterranean	Therophyte	*Pl.we*	A
*Poa bulbosa*	Poaceae	Paleotemperate	Hemicryptophyte	*Po.bu*	A
*Polygala venulosa*	Polygalaceae	Mediterranean	Hemicryptophyte	*Po.ve*	A
*Reichardia picroides*	Asteraceae	Mediterranean	Hemicryptophyte	*Re.pi*	Β
Reseda alba *	Resedaceae	Mediterranean	Therophyte	*Re.al*	Β
*Satureja thymbra*	Lamiaceae	Mediterranean	Chamaephyte	*Sa.th*	A
*Scolymus hispanicus*	Asteraceae	Mediterranean	Hemicryptophyte	*Sc.hi*	Β
Scorzonera crocifolia *	Asteraceae	Greek endemic	Hemicryptophyte	*Sc.cr*	A, B
*Silene colorata*	Caryophyllaceae	Mediterranean	Therophyte	*Si.co*	Β
*Silene conica*	Caryophyllaceae	European-SW Asian	Therophyte	*Si.co*	A
Silene corinthiaca *	Caryophyllaceae	Greek endemic	Therophyte	*Si.co*	B
*Teucrium capitatum*	Lamiaceae	Mediterranean	Chamaephyte	*Te.ca*	A
*Thesium bergeri*	Santalaceae	Mediterranean	Hemicryptophyte	*Th.be*	A
Thymelaea tartonraira *	Thymelaeaceae	Mediterranean	Phanerophyte	*Th.ta*	A
Thymbra capitata *	Lamiaceae	Mediterranean	Chamaephyte	*Th.ca*	A
*Tordylium apulum*	Apiaceae	Mediterranean	Therophyte	*To.ap*	Β
*Tragopogon porrifolius*	Asteraceae	Mediterranean	Hemicryptophyte	*Tr.po*	Β
*Trifolium stellatum*	Fabaceae	Mediterranean	Therophyte	*Tr.st*	Β
*Trigonella spruneriana*	Fabaceae	Mediterranean	Therophyte	*Tr.sp*	B
*Tuberaria guttata*	Cistaceae	Mediterranean-Atlantic	Therophyte	*Tu.gu*	A
*Urospermun picroides*	Asteraceae	Mediterranean	Therophyte	*Ur.pi*	Β
*Valantia hispida*	Rubiaceae	Mediterranean	Therophyte	*Va.hi*	Β
*Vulpia ciliata*	Poaceae	Mediterranean-SW Asian	Therophyte	*Vu.ci*	A

**Table 4:** Concentrations of potentially toxic elements in aerial plant parts (mg kg^−1^ dry weight). Plant code definitions are provided in Table 3. Data are presented as mean ± standard deviation (n = 3).

Plant Code	As	Cd	Co	Cr	Cu	Fe	Mg	Mn	Ni	Pb	Sb	Zn
**Site A (mining site)**												
As.lu	**3.13** ± 0.45 ^a^	**6.71** ± 0.52 ^abcd^	0.24 ± 0.06 ^ab^	**4.24** ± 0.55 ^fg^	3.73 ± 0.07 ^abc^	**589.46** ± 87.71 ^cde^	1262.65 ± 7.74 ^cd^	16.40 ± 0.35 ^abcde^	**2.42** ± 0.39 ^cde^	**241.71** ± 9.67 ^bcd^	**2.70** ± 0.10 ^c^	**575.54** ± 73.52 ^c^
Br.re.A	**3.15** ± 0.44 ^a^	**2.86** ± 0.48 ^abc^	**1.08** ± 1.40 ^b^	**5.64** ± 0.07 ^g^	3.95 ± 0.20 ^abc^	**523.88** ± 87.40 ^bcd^	765.15 ± 24.94 ^abc^	20.04 ± 1.30 ^bcde^	**4.04** ± 1.22 ^f^	**153.55** ± 12.21 ^abc^	**1.46** ± 0.20 ^abc^	**254.84** ± 32.29 ^ab^
Ce.la	**1.54** ± 0.14 ^a^	**1.35** ± 0.06 ^ab^	**1.00** ± 0.01 ^a^	**1.18** ± 0.21 ^abcd^	5.38 ± 0.43 ^cd^	146.96 ± 68.23 ^a^	1411.22 ± 10.79 ^cde^	7.89 ± 0.74 ^abc^	1.07 ± 0.06 ^abc^	**61.44** ± 19.38 ^ab^	0.97 ± 0.22 ^abc^	**269.84** ± 54.86 ^ab^
Ci.cr	**1.06** ± 0.21 ^a^	**16.13** ± 3.45 ^bcde^	0.17 ± 0.03 ^a^	**1.57** ± 0.51 ^abcde^	4.25 ± 0.36 ^abc^	225.26 ± 80.48 ^abc^	1708.07 ± 215.51 ^def^	18.07 ± 3.92 ^abcde^	1.40 ± 0.25 ^abc^	**47.13** ± 9.09 ^ab^	0.90 ± 0.18 ^abc^	**252.42** ± 25.40 ^ab^
Ci.sa	**1.42 ± 0.19 ^a^**	**18.40** ± 0.94 ^de^	0.17 ± 0.03 ^a^	**1.38** ± 0.11 ^abcde^	2.53 ± 0.13 ^abc^	233.44 ± 2.17 ^abc^	1380.04 ± 96.22 ^cde^	30.31 ± 1.92 ^e^	**3.28** ± 0.03 ^ef^	**49.14** ± 2.21 ^ab^	0.87 ± 0.25 ^abc^	**272.02** ± 17.40 ^ab^
Di.di.A	**2.34** ± 0.34 ^a^	**12.71** ± 0.51 ^abcde^	0.18 ± 0.02 ^a^	**1.44** ± 0.26 ^abcde^	3.62 ± 0.26 ^abc^	226.91 ± 41.24 ^abc^	1848.24 ± 113.41 ^def^	29.39 ± 2.43 ^de^	1.76 ± 0.20 ^bcde^	**80.34** ± 22.28 ^abc^	**1.57** ± 0.14 ^abc^	**619.66** ± 29.76 ^c^
Fu.th	**1.09** ± 0.21 ^a^	**5.56** ± 0.195 ^abcd^	0.4 ± 0.01 ^a^	**2.00** ± 0.09 ^cde^	2.88 ± 0.05 ^abc^	221.22 ± 15.47 ^abc^	882.37 ± 11.17 ^bc^	11.35 ± 0.40 ^abcd^	1.71 ± 0.07 ^bcd^	**192.30** ± 15.38 ^abc^	**1.12** ± 0.18 ^abc^	**170.63** ± 4.17 ^ab^
He.st	**6.24** ± 2.10 ^a^	**16.74** ± 13.61 ^cde^	0.24 ± 0.16 ^ab^	**2.62** ± 2.25 ^de^	7.01 ± 5.43 ^ab^	**612.99** ± 524.76 ^de^	817.28 ± 683.69 ^abc^	23.90 ± 20.21 ^cde^	**2.40** ± 1.90 ^cde^	**463.60** ± 389.08 ^d^	**2.66** ± 1.714b ^c^	**573.69** ± 421.58 ^c^
Kn.in.A	**1.16** ± 0.33 ^a^	**1.47** ± 0.05 ^ab^	0.07 ± 0.02 ^a^	0.94 ± 0.14 ^abc^	3.66 ± 0.02 ^abc^	95.92 ± 13.98 ^a^	2116.39 ± 21.07 ^ef^	9.83 ± 0.05 ^abc^	1.33 ± 0.11 ^abc^	**33.57** ± 7.50 ^ab^	0.88 ± 0.21 ^abc^	**218.40** ± 9.20 ^ab^
Ph.gr.A	**1.17** ± 0.49 ^a^	**25.54** ± 10.96 ^e^	0.27 ± 0.13 ^ab^	**1.20** ± 1.09 ^abcd^	7.06 ± 5.47 ^ab^	**252.02** ± 27.34 ^abcd^	1255.98 ± 1063.72 ^cd^	25.12 ± 21.78 ^cde^	1.12 ± 0.84 ^abc^	**17.28** ± 14.93 ^ab^	0.54 ± 0.34 ^abc^	**240.75** ± 18.92 ^ab^
Sc.cr	**0.95** ± 0.16 ^a^	**19.39** ± 1.76 ^de^	0.06 ± 0.03 ^a^	0.36 ± 0.03 ^ab^	6.81 ± 0.49 ^ab^	46.39 ± 4.44 ^a^	2386.17 ± 214.35 ^f^	23.58 ± 4.32 ^a^	1.20 ± 0.19 ^a^	**6.64** ± 0.10 ^a^	0.87 ± 0.00 ^abc^	**145.55** ± 6.87 ^ab^
Th.ca	**1.06** ± 0.22 ^a^	0 ± 0.00 ^a^	0.11 ± 0.01 ^a^	**1.23** ± 0.03 ^abcd^	3.57 ± 0.36 ^abc^	162.55 ± 25.83 ^ab^	1456.08 ± 237.19 ^cde^	7.29 ± 0.51 ^abc^	1.10 ± 0.09 ^abc^	**29.53** ± 0.86 ^ab^	0.78 ± 0.03 ^abc^	55.84 ± 9.44 ^ab^
Th.ta	**1.25** ± 0.29 ^a^	**2.25** ± 0.23 ^abc^	0.15 ± 0.03 ^a^	**1.69** ± 0.01 ^bcde^	3.65 ± 0.03 ^abc^	**269.45** ± 45.92 ^abcd^	1347.18 ± 143.83 ^cde^	8.19 ± 0.73 ^abc^	1.14 ± 0.01 ^abc^	**24.79** ± 0.65 ^ab^	0.75 ± 0.11 ^abc^	58.83 ± 5.45 ^ab^
**Site B (metallurgical site)**												
Ai.el	**2.44** ± 0.46 ^a^	0.13 ± 0.01 ^a^	0.07 ± 0.00 ^a^	0.02 ± 0.01 ^a^	0.26 ± 0.02 ^a^	2.98 ± 0.42 ^a^	13.60 ± 0.26 ^a^	0.21 ± 0.01 ^a^	0.07 ± 0.02 ^a^	2.09 ± 0.18 ^a^	0.41 ± 0.05 ^abc^	8.32 ± 0.48 ^a^
Al.ti	**31.02** ± 7.02 ^b^	**4.37** ± 0.01 ^abcd^	0.27 ± 0.02 ^ab^	**2.86** ± 0.04 ^ef^	11.22 ± 0.28 ^d^	**930.81** ± 169.36 ^e^	1738.68 ± 11.17 ^def^	52.72 ± 3.44 ^f^	**3.08** ± 0.01 ^def^	**304.89** ± 26.06 ^cd^	**6.75** ± 3.42 ^d^	**336.88** ± 0.22 ^bc^
An.vu	**1.44** ± 0.29 ^a^	0.11 ± 0.01 ^a^	0.08 ± 0.04 ^a^	0.02 ± 0.00 ^a^	0.28 ± 0.01 ^a^	1.43 ± 0.18 ^a^	32.92 ± 1.413 ^a^	0.29 ± 0.00 ^a^	0.10 ± 0.02 ^a^	0.71 ± 0.13 ^a^	0.42 ± 0.13 ^abc^	12.54 ± 0.30 ^a^
Ba.ac	**2.11** ± 0.38 ^a^	0.06 ± 0.01 ^a^	0.09 ± 0.01 ^a^	0.01 ± 0.01 ^a^	0.53 ± 0.05 ^a^	6.23 ± 2.06 ^a^	46.49 ± 4.01 ^a^	0.37 ± 0.07 ^a^	0.11 ± 0.04 ^a^	0.80 ± 0.15 ^a^	0.24 ± 0.09 ^a^	5.56 ± 1.76 ^a^
Br.re.B	**1.17** ± 0.27 ^a^	0.07 ± 0.01 ^a^	0.08 ± 0.05 ^a^	0.02 ± 0.00 ^a^	0.26 ± 0.05 ^a^	4.99 ± 1.58 ^a^	9.51 ± 1.68 ^a^	0.33 ± 0.06 ^a^	0.134 ± 0.01 ^a^	0.42 ± 0.26 ^a^	0.62 ± 0.14 ^abc^	11.12 ± 1.80 ^a^
Da.vi	**0.93** ± 0.10 ^a^	0.07 ± 0.01 ^a^	0.07 ± 0.05 ^a^	0.02 ± 0.01 ^a^	0.22 ± 0.02 ^a^	0.51 ± 0.05 ^a^	11.28 ± 0.07 ^a^	0.18 ± 0.01 ^a^	0.06 ± 0.03 ^a^	0.54 ± 0.31 ^a^	0.33 ± 0.10 ^a^	4.41 ± 0.19 ^a^
Di.di.B	**2.88** ± 1.66 ^a^	**2.24** ± 0.42 ^abc^	0.11 ± 0.02 ^a^	0.86 ± 0.42 ^abc^	2.72 ± 0.59 ^abc^	151.76 ± 84.70 ^ab^	1543.46 ± 330.79 ^cde^	23.23 ± 7.88 ^cde^	1.27 ± 0.66 ^abc^	**14.62** ± 8.16 ^ab^	0.85 ± 0.10 ^abc^	98.27 ± 39.26 ^ab^
Gl.fl	**2.48** ± 0.85 ^a^	0.10 ± 0.01 ^a^	0.11 ± 0.05 ^a^	0.60 ± 0.37 ^abc^	3.44 ± 0.62 ^abc^	40.78 ± 20.56 ^a^	191.43 ± 33.56 ^ab^	0.90 ± 0.28 ^a^	0.44 ± 0.22 ^ab^	1.09 ± 0.26 ^a^	0.57 ± 0.13 ^abc^	28.08 ± 3.87 ^a^
Kn.in.B	**0.78** ± 0.40 ^a^	0.07 ± 0.01 ^a^	0.05 ± 0.04 ^a^	0.03 ± 0.01 ^a^	0.34 ± 0.01 ^a^	1.25 ± 0.33 ^a^	42.24 ± 1.84 ^a^	0.16 ± 0.02 ^a^	0.08 ± 0.01 ^a^	0.27 ± 0.12 ^a^	0.43 ± 0.10 ^abc^	12.40 ± 0.34 ^a^
La.tu	**1.19** ± 0.12 ^a^	0.34 ± 0.02 ^a^	0.10 ± 0.05 ^a^	0.02 ± 0.01 ^a^	0.567 ± 0.01 ^a^	2.64 ± 0.79 ^a^	60.14 ± 1.68 ^a^	0.34 ± 0.01 ^a^	0.07 ± 0.03 ^a^	1.22 ± 0.21 ^a^	0.29 ± 0.26 ^a^	6.12 ± 0.20 ^a^
Ph.gr.B	**1.77** ± 0.18 ^a^	0.20 ± 0.01 ^a^	0.08 ± 0.01 ^a^	0.02 ± 0.01 ^a^	0.70 ± 0.03 ^a^	4.03 ± 0.59 ^a^	23.37 ± 1.51 ^a^	0.47 ± 0.06 ^a^	0.12 ± 0.04 ^a^	0.62 ± 0.04 ^a^	0.43 ± 0.01 ^abc^	3.62 ± 0.09 ^a^
Re.al.B1	**2.57** ± 0.46 ^a^	0.09 ± 0.02 ^a^	0.05 ± 0.02 ^a^	0.03 ± 0.01 ^a^	0.27 ± 0.01 ^a^	4.98 ± 0.10 ^a^	45.04 ± 1.45 ^a^	0.587 ± 0.03 ^a^	0.160 ± 0.03 ^a^	0.916 ± 0.09 ^a^	0.34 ± 0.10 ^ab^	11.585 ± 3.74 ^a^
Re.al.B2	**6.59** ± 7.62 ^a^	0.21 ± 0.06 ^a^	0.10 ± 0.02 ^a^	0.05 ± 0.04 ^a^	0.55 ± 0.09 ^a^	9.42 ± 1.22 ^a^	41.56 ± 0.68 ^a^	0.31 ± 0.06 ^a^	0.12 ± 0.05 ^a^	1.56 ± 1.38 ^a^	0.59 ± 0.27 ^abc^	27.61 ± 3.89 ^a^
Sc.cr	**1.04** ± 0.11 ^a^	0.16 ± 0.01 ^a^	0.10 ± 0.02 ^a^	0.05 ± 0.02 ^a^	0.41 ± 0.05 ^a^	2.04 ± 1.06 ^a^	49.15 ± 10.89 ^a^	0.84 ± 0.27 ^cde^	0.12 ± 0.03 ^abc^	0.35 ± 0.09 ^a^	0.36 ± 0.24 ^ab^	4.38 ± 1.27 ^a^
Si.co	**4.75** ± 1.53 ^a^	0.07 ± 0.00 ^a^	0.10 ± 0.05 ^a^	0.25 ± 0.15 ^ab^	0.76 ± 0.24 ^ab^	7.47 ± 6.67 ^a^	44.84 ± 4.51 ^a^	2.08 ± 1.36 ^ab^	0.12 ± 0.06 ^a^	2.48 ± 1.62 ^a^	0.32 ± 0.07 ^a^	64.42 ± 42.02 ^ab^
*Normal concentrations* ‡	0.02−0.1	0.1−0.5	0.05−0.5	0.02−1.0	5.0−20.0	50.0−250.0	1000.0–5000.0	20.0−200.0	0.2−2.0	1.0−5.0	<1.0	20.0−100.0

‡ Typical plant tissue concentration ranges [34]. Values exceeding these normal concentrations and considered excessive or toxic for most plants are marked boldface. Different letters within a column indicate significant differences among species (*p* < 0.05).

**Table 5:** Bioconcentration factors of native plant species for the assessed potential toxic elements. The meaning of plant codes can be found in Table 3. The data present means ± SD.

Code	As	Cd	Co	Cr	Cu	Fe	Mn	Ni	Pb	Sb	**Zn**
**Site A (mining site)**											
As.lu	0.0063 ± 0.0009 ^a^	0.0485 ± 0.0038 ^abcd^	0.018 ± 0.0045 ^ab^	0.0119 ± 0.0015 ^ef^	0.0221 ± 0.0004 ^abc^	0.0150 ± 0.0022 ^de^	0.0091 ± 0.0002 ^abcd^	0.0127 ± 0.002 ^ef^	0.0089 ± 0.0004 ^bcd^	0.0097 ± 0.0004 ^c^	0.0300 ± 0.0038 ^c^
Br.re.A	0.0063 ± 0.0008 ^a^	0.0207 ± 0.0035 ^abc^	0.0824 ± 0.1067 ^b^	0.0157 ± 0.0002 ^f^	0.0234 ± 0.0011 ^abc^	0.0133 ± 0.0022 ^bcde^	0.0111 ± 0.0007 ^bcd^	0.0201 ± 0.006 ^g^	0.0056 ± 0.0004 ^abc^	0.0052 ± 0.0007 ^abc^	0.0132 ± 0.0016 ^ab^
Ce.la	0.003 ± 0.0002 ^a^	0.0097 ± 0.0004 ^ab^	0.0073 ± 0.001 ^a^	0.0033 ± 0.0005 ^abcd^	0.0319 ± 0.0025 ^bc^	0.0037 ± 0.0017 ^a^	0.0043 ± 0.0004 ^ab^	0.0053 ± 0.0003 ^abcde^	0.0022 ± 0.0007 ^ab^	0.0034 ± 0.0007 ^abc^	0.014 ± 0.0028 ^ab^
Ci.cr	0.0021 ± 0.0004 ^a^	0.1168 ± 0.0249 ^bcde^	0.0127 ± 0.002 ^a^	0.0044 ± 0.0014 ^abcd^	0.0252 ± 0.0021 ^abc^	0.0057 ± 0.002 ^abc^	0.01 ± 0.0021 ^abcd^	0.0069 ± 0.0012 ^abcde^	0.0017 ± 0.0003 ^ab^	0.0032 ± 0.0006 ^abc^	0.0131 ± 0.0013 ^ab^
Ci.sa	0.0028 ± 0.0003 ^a^	0.1333 ± 0.0068 ^de^	0.0132 ± 0.0022 ^a^	0.0038 ± 0.0003 ^abcd^	0.015 ± 0.0007 ^abc^	0.0059 ± 0.0 ^abcd^	0.0168 ± 0.001 ^d^	0.0163 ± 0.0001 ^fg^	0.0018 ± 0.0 ^ab^	0.0031 ± 0.0008 ^abc^	0.0141 ± 0.0009 ^ab^
Di.di. A	0.0046 ± 0.0006 ^a^	0.092 ± 0.0036 ^abcde^	0.014 ± 0.0014 ^a^	0.004 ± 0.0007 ^abcd^	0.0214 ± 0.0015 ^abc^	0.0057 ± 0.001 ^abcd^	0.0163 ± 0.0013 ^cd^	0.0087 ± 0.0009 ^def^	0.0029 ± 0.0008 ^ab^	0.0056 ± 0.0005 ^abc^	0.0322 ± 0.0015 ^c^
Fu.th	0.0022 ± 0.0004 ^a^	0.0402 ± 0.0014 ^abcd^	0.0103 ± 0.0007 ^a^	0.0055 ± 0.0002 ^bcd^	0.0171 ± 0.0002 ^abc^	0.0056 ± 0.0003 ^abc^	0.0063 ± 0.0002 ^abc^	0.0085 ± 0.0003 ^bcdef^	0.0071 ± 0.0005 ^abc^	0.004 ± 0.0006 ^abc^	0.0088 ± 0.0002 ^ab^
He.st	0.0125 ± 0.0042 ^a^	0.1212 ± 0.0985 ^cde^	0.0181 ± 0.0123 ^ab^	0.0073 ± 0.0063 ^de^	0.0416 ± 0.0322 ^c^	0.0156 ± 0.0133 ^e^	0.0132 ± 0.0112 ^bcd^	0.0119 ± 0.0094 ^ef^	0.0171 ± 0.0143 ^d^	0.0095 ± 0.0061 ^bc^	0.0298 ± 0.0219 ^c^
Kn.in.A	0.0022 ± 0.0006 ^a^	0.0106 ± 0.0003 ^ab^	0.005 ± 0.0014 ^a^	0.0026 ± 0.0003 ^abcd^	0.0217 ± 0.0001 ^abc^	0.0024 ± 0.0003 ^a^	0.0054 ± 0.0 ^ab^	0.0066 ± 0.0005 ^abcde^	0.0012 ± 0.0002 ^ab^	0.0031 ± 0.0007 ^abc^	0.0113 ± 0.0004 ^ab^
Ph.gr.A	0.0023 ± 0.0009 ^a^	0.1849 ± 0.1518 ^e^	0.0203 ± 0.0096 ^ab^	0.0033 ± 0.003 ^abcd^	0.0419 ± 0.0325 ^c^	0.0064 ± 0.0058 ^abcde^	0.0139 ± 0.0121 ^bcd^	0.0055 ± 0.0041 ^abcde^	0.0006 ± 0.0005 ^ab^	0.0019 ± 0.0012 ^a^	0.0125 ± 0.0095 ^ab^
Sc.cr.A	0.0019 ± 0.0003 ^a^	0.1405 ± 0.0128 ^de^	0.0047 ± 0.0019 ^a^	0.001 ± 0.0001 ^a^	0.0404 ± 0.003 ^c^	0.0012 ± 0.0001 ^a^	0.0131 ± 0.0024 ^bcd^	0.006 ± 0.001 ^abcde^	0.0002 ± 0.0 ^a^	0.0031 ± 0.0000 ^abc^	0.0076 ± 0.0004 ^ab^
Th.ca	0.0021 ± 0.0004 ^a^	0.0042 ± 0.0006 ^a^	0.0084 ± 0.0007 ^a^	0.0034 ± 0.0 ^abcd^	0.0212 ± 0.0021 ^abc^	0.0041 ± 0.0006 ^ab^	0.004 ± 0.0002 ^ab^	0.0054 ± 0.0004 ^abcde^	0.001 ± 0.0 ^ab^	0.0028 ± 0.0001 ^ab^	0.0029 ± 0.0004 ^a^
Th.ta	0.0025 ± 0.000 ^3 a^	0.0163 ± 0.0011 ^a^	0.0116 ± 0.0012 ^a^	0.0047 ± 0.0 ^abcd^	0.0216 ± 0.0001 ^abc^	0.0068 ± 0.0007 ^abcde^	0.0045 ± 0.0002 ^ab^	0.0056 ± 0.0000 ^abcde^	0.0009 ± 0.0 ^ab^	0.0026 ± 0.0002 ^a^	0.003 ± 0.0001 ^a^
**Site B (metallurgical site)**											
Ai. el	0.0007 ± 0.0001 ^a^	0.0009 ± 0.0 ^a^	0.0094 ± 0.0005 ^a^	0.0001 ± 0.0 ^a^	0.0003 ± 0.0 ^a^	−	−	0.0004 ± 0.0001 ^a^	−	0.0011 ± 0.0001 ^a^	0.0004 ± 0.0 ^a^
Al. ti	0.0722 ± 0.0632 ^b^	0.0329 ± 0.0007 ^abcd^	0.0358 ± 0.0025 ^ab^	0.0224 ± 0.0003 ^g^	0.0154 ± 0.0003 ^abc^	0.0147 ± 0.0026 ^cde^	0.0178 ± 0.0011 ^d^	0.0209 ± 0.0 ^g^	0.0117 ± 0.001 ^cd^	0.0182 ± 0.0092 ^d^	0.02 ± 0.0 ^bc^
An.vu	0.0004 ± 0.0 ^a^	0.0008 ± 0.0 ^a^	0.0111 ± 0.0045 ^a^	0.0001 ± 0.0 ^a^	0.0003 ± 0.0 ^a^	−	−	0.0006 ± 0.0001 ^ab^	−	0.0011 ± 0.0003 ^a^	0.0007 ± 0.0 ^a^
Ba.ac	0.0006 ± 0.0001 ^a^	0.0004 ± 0.0 ^a^	0.0112 ± 0.001 ^a^	−	0.0007 ± 0.0 ^a^	−	0.0001 ± 0.0 ^a^	0.0007 ± 0.0002 ^ab^	−	0.0006 ± 0.0002 ^a^	0.0003 ± 0.0001 ^a^
Br.re.B	0.0003 ± 0.0 ^a^	0.0005 ± 0.0 ^a^	0.0109 ± 0.0064 ^a^	0.0001 ± 0.0 ^a^	0.0003 ± 0.0 ^a^	−	0.0001 ± 0.0 ^a^	0.0009 ± 0.0 ^abcd^	−	0.0016 ± 0.0003 ^a^	0.0006 ± 0.0001 ^a^
Da.vi	0.0002 ± 0.0 ^a^	0.0005 ± 0.0 ^a^	0.0092 ± 0.0069 ^a^	0.0001 ± 0.0 ^a^	0.0003 ± 0.0 ^a^	−	−	0.0003 ± 0.0002 ^a^	−	0.0008 ± 0.0002 ^a^	0.0002 ± 0.0 ^a^
Di.di.B	0.0008 ± 0.0004 ^a^	0.0168 ± 0.0032 ^abc^	0.0143 ± 0.003 ^a^	0.0067 ± 0.0032 ^cde^	0.0037 ± 0.0008 ^a^	0.0024 ± 0.0013 ^a^	0.0078 ± 0.0026 ^abcd^	0.0086 ± 0.0045 ^cdef^	0.0005 ± 0.0003 ^ab^	0.0023 ± 0.0002 ^a^	0.0058 ± 0.0023 ^ab^
Gl.fl	0.0007 ± 0.0002 ^a^	0.0007 ± 0.0 ^a^	0.0142 ± 0.0063 ^a^	0.0047 ± 0.0029 ^abcd^	0.0047 ± 0.0008 ^ab^	0.0006 ± 0.0003 ^a^	0.0003 ± 0.0 ^a^	0.003 ± 0.0015 ^abcd^	−	0.0015 ± 0.0003 ^a^	0.0016 ± 0.0002 ^a^
Kn.in.B	0.0002 ± 0.0001 ^a^	0.0005 ± 0.0 ^a^	0.0061 ± 0.0049 ^a^	0.0002 ± 0.0001 ^a^	0.0004 ± 0.0 ^a^	−	−	0.0005 ± 0.0 ^a^	−	0.0011 ± 0.0002 ^a^	0.0007 ± 0.0 ^a^
La. tu	0.0003 ± 0.0 ^a^	0.0025 ± 0.0001 ^a^	0.0131 ± 0.0064 ^a^	0.0001 ± 0.0001 ^a^	0.0007 ± 0.0 ^a^	−	0.0001 ± 0.0 ^a^	0.0005 ± 0.0001 ^a^	−	0.0007 ± 0.0007 ^a^	0.0003 ± 0.0 ^a^
Ph.gr.B	0.0005 ± 0.0 ^a^	0.0014 ± 0.0 ^a^	0.0109 ± 0.0016 ^a^	0.0001 ± 0.0 ^a^	0.0009 ± 0.0 ^a^	−	0.0001 ± 0.0 ^a^	0.0008 ± 0.0003 ^abc^	−	0.0011 ± 0.0 ^a^	0.0002 ± 0.0 ^a^
Re.al.B1	0.0007 ± 0.0001 ^a^	0.0007 ± 0.0001 ^a^	0.007 ± 0.0030 ^a^	0.0002 ± 0.0 ^a^	0.0003 ± 0.0 ^a^	−	0.0001 ± 0.0 ^a^	0.001 ± 0.0002 ^abcd^	−	0.0009 ± 0.0002 ^a^	0.0006 ± 0.0002 ^a^
Re.al.B2	0.0019 ± 0.0018 ^a^	0.0016 ± 0.0003 ^a^	0.0127 ± 0.0019 ^a^	0.0004 ± 0.0002 ^a^	0.0007 ± 0.0001 ^a^	0.0001 ± 0.0 ^a^	0.0001 ± 0.0 ^a^	0.0008 ± 0.0002 ^abc^	−	0.0015 ± 0.0005 ^a^	0.0016 ± 0.0001 ^a^
Sc.cr.B	0.0003 ± 0.0 ^a^	0.0012 ± 0.0 ^a^	0.0125 ± 0.0027 ^a^	0.0003 ± 0.0001 ^ab^	0.0005 ± 0.0 ^a^	−	0.0002 ± 0.0 ^a^	0.0008 ± 0.0001 ^abc^	−	0.0009 ± 0.0006 ^a^	0.0002 ± 0.0 ^a^
Si.co	0.0013 ± 0.0004 ^a^	0.0005 ± 0.0 ^a^	0.0129 ± 0.0061 ^a^	0.0019 ± 0.0011 ^abc^	0.001 ± 0.0003 ^a^	0.0001 ± 0.0001 ^a^	0.0007 ± 0.0004 ^a^	0.0008 ± 0.0004 ^abc^	−	0.0008 ± 0.0001 ^a^	0.0038 ± 0.0024 ^a^

Different letters within a column indicate significant differences between species (*p* < 0.05).

## Data Availability

Data are contained within the article; further inquiries can be directed to the corresponding author.
